# The ERα-NRF2 signalling axis promotes bicalutamide resistance in prostate cancer

**DOI:** 10.1186/s12964-022-00979-0

**Published:** 2022-11-14

**Authors:** Lei Tian, Yanfei Peng, Kuo Yang, Jiasong Cao, Xiaoling Du, Zhixian Liang, Jiandang Shi, Ju Zhang

**Affiliations:** 1 Department of Biochemistry and Molecular Biology, College of Life Sciences, Bioactive Materials Key Lab of the Ministry of Education, Nankai University, Tianjin, 300071 China; 2 School of Integrative Medicine, Tianjin University of Traditional Chinese Medicine, Tianjin, 301617 China; 3 Department of Urology, The Second Hospital of Tianjin Medical University, Tianjin, 300211 China; 4 School of Biomedical Sciences, The Chinese University of Hong Kong, Hong Kong, 999077 China

**Keywords:** Bicalutamide, ERα, NRF2, Castration-resistant, PCa

## Abstract

**Background:**

Bicalutamide is a nonsteroidal antiandrogen widely used as a first-line clinical treatment for advanced prostate cancer (PCa). Although patients initially show effective responses to bicalutamide treatment, resistance to bicalutamide frequently occurs and leads to the development of castration-resistant PCa (CRPC). This research investigated the roles of the oestrogen receptor α (ERα)-nuclear factor E2-related factor 2 (NRF2) signalling pathway in bicalutamide resistance in PCa cells.

**Methods:**

We performed bioinformatic analysis and immunohistochemical staining on normal and cancerous prostate tissue to evaluate ERα and NRF2 expression and their correlation. Gene expression and localization in PCa cell lines were further investigated using real-time reverse transcription PCR/Western blotting and immunofluorescence staining. We treated PCa cells with the ER inhibitor tamoxifen and performed luciferase reporter assays and chromatin immunoprecipitation (ChIP) assays to understand ERα-dependent NRF2 expression. Overexpression and knockdown of ERα and NRF2 were used to explore the potential role of the ERα-NRF2 signalling axis in bicalutamide resistance in PCa cells.

**Results:**

We found that the expression of ERα and NRF2 was positively correlated and was higher in human CRPC tissues than in primary PCa tissues. Treatment with oestrogen or bicalutamide increased the expression of ERα and NRF2 as well as NRF2 target genes in PCa cell lines. These effects were blocked by pretreatment with tamoxifen. ChIP assays demonstrated that ERα directly binds to the oestrogen response element (ERE) in the NRF2 promoter. This binding led to increased transcriptional activity of NRF2 in a luciferase reporter assay. Activation of the ERα-NRF2 signalling axis increased the expression of bicalutamide resistance-related genes. Inhibition of this signalling axis by knockdown of ERα or NRF2 downregulated the expression of bicalutamide resistance-related genes and inhibited the proliferation and migration of PCa cells.

**Conclusions:**

We demonstrated the transcriptional interaction between ERα and NRF2 in CRPC tissues and cell lines by showing the direct binding of ERα to the ERE in the NRF2 promoter under oestrogen treatment. Activation of the ERα-NRF2 signalling axis contributes to bicalutamide resistance in PCa cells, suggesting that the ERα-NRF2 signalling axis is a potential therapeutic target for CRPC.

**Video Abstract**

**Supplementary Information:**

The online version contains supplementary material available at 10.1186/s12964-022-00979-0.

## Background

Prostate cancer (PCa) is the second most common cancer and the fifth leading cause of cancer-related death in men [1]. Androgen deprivation therapy (ADT) is the initial treatment for most primary PCa patients [2]. Initial ADT involves surgical castration (for example, bilateral orchiectomy) and chemical castration to remove circulating serum testosterone produced by the testes [3, 4]. However, the persistence of androgen receptor (AR) signalling axis activity, especially the production of androgens in tumours, during systemic castration undermines the therapeutic efficacy of ADT. Bicalutamide is a second-generation AR antagonist that is designed to prevent androgens from binding to AR and is widely used in prostate cancer patients. Although PCa are initially responsive to bicalutamide, they become refractory and later acquire resistance, which leads to the development of incurable castration-resistant PCa (CRPC) [5, 6]. The molecular mechanisms related to bicalutamide resistance in CRPC are not yet understood.

Bonkhoff et al. [7] first described the presence of oestrogen receptor α (ERα) in premalignant and advanced PCa in 1999. The effects of oestrogen appear to be related to the progression of CRPC. The highest ERα mRNA and protein expression has been observed in metastatic lesions and CRPC tissues, and there are a large number of oestrogen-responsive cells in these tumours [8]. Previous reports have shown that advanced PCa cells can switch from AR signalling to ERα signalling in response to ADT to maintain tumour cell proliferation under androgen-deprived conditions [9]. We previously found that 17β-oestradiol (E2) promoted tumour formation and metastasis in castrated mice bearing PC3 xenografts and that the ERα–NOTCH1 signalling axis enhanced the basal stem-like phenotype and epithelial-mesenchymal transition in PCa cells [10]. Furthermore, we found that aromatase expression was increased in CRPC cell lines and tissues, whereas the abundant aromatase increased the concentration of endogenous oestrogen metabolized by testosterone, and that ERα transcriptional activity was enhanced. Treatment with bicalutamide can upregulate the expression of aromatase, leading to CRPC progression [11], suggesting the role of ER signalling pathways in bicalutamide resistance. Understanding the effects of oestrogen on the development of resistance to bicalutamide treatment, which contributes to the progression of CRPC, is urgently needed to facilitate novel therapeutic development. We used mRNA sequencing (mRNA-seq) to compare bicalutamide-resistant cell lines to their parental cell lines to identify the potential oestrogen pathways that contribute to resistance and found that the nuclear factor E2-related factor 2 (NRF2) pathway is linked to bicalutamide resistance in PCa.

NRF2 is a pivotal transcription factor that induces the expression of several antioxidant enzymes to protect against oxidative-induced cell damage. However, the role of NRF2 in PCa is still ambiguous. On the one hand, ADT increases the levels of reactive oxygen species (ROS), which may activate AR signalling. Overexpression or activation of NRF2 may decrease ROS levels to inhibit the transition of PCa to CRPC [12, 13]. On the other hand, both ROS-dependent and ROS-independent NRF2 activation have been reported to induce chemoresistance in PCa cells [14, 15].

The regulatory role of ERα in the NRF2 signalling pathway seems to be controversial. A previous report indicated that oestrogen can increase NRF2 activity in MCF-7 breast cancer cells via activation of the PI3K pathway [16]. Meng et al. [17] reported that in a cerebral ischaemia‒reperfusion model, notoginsenoside R1 (NGR1) exhibited a neuroprotective effect by increasing the expression of ERα and ERβ and subsequent protein kinase B (Akt)/Nrf2 pathway activation. Similar results were observed in a diabetic cardiomyopathy model. The expression of ERα was upregulated by NGR1 treatment and subsequently promoted nuclear translocation of Nrf2 to execute cardioprotective effects [18]. In human umbilical cord blood mesenchymal stem cells, E2 can induce nuclear NRF2 expression via interaction with ERα, which protects cells against high glucose-induced mitochondrial ROS production and autophagic cell death [19]. Recent research reported that silibinin activates antioxidative NRF2 pathways in pancreatic β cells through upregulation of ERα expression [20]. Collectively, these reports suggest that ERα promotes the activation of the NRF2 signalling pathway. However, controversial results also exist. Ansell et al. [21] reported that E2-bound ERα interacts with Nrf2 to repress Nrf2-mediated transcription. The regulatory role of ERα in the expression and activation of NRF2 in the prostate has not been well elucidated. Furthermore, the literature indicates that ERα may indirectly activate the NRF2 signalling pathway by promoting its nuclear translocation. However, we found that ERα directly binds to the oestrogen response element (ERE) in the NRF2 promoter to enhance gene transcription.

In this research, we used mRNA sequencing (mRNA-seq) to compare bicalutamide-resistant cell lines to their parental cell lines to identify the potential oestrogen pathways that contribute to bicalutamide resistance. We further explored the regulatory mechanism mediated by ERα to understand the biology of bicalutamide resistance in PCa and to identify the ERα-NRF2 signalling pathway as a potential therapeutic target.

## Materials and methods

### Cell culture and bicalutamide treatment

Androgen-independent LNCaP abl PCa cells (a kind gift from Helmut Klocker; Department of Urology, Medical University of Innsbruck, Innsbruck, Austria) were cultured in RPMI-1640 medium (Sigma, St. Louis, MO, USA) supplemented with 1% penicillin/streptomycin (P/S; HyClone, Logan, UT, USA) and 10% charcoal dextran stripped foetal bovine serum (CDS-FBS; Invitrogen, Carlsbad, CA). LNCaP and PC3 PCa cells were cultured in RPMI 1640 medium (Gibco, Grand Island, NY, USA) supplemented with 1% P/S and 10% FBS (Gibco). The characteristics and culture methods of these cell lines are described in Additional file 1. LNCaP and LNCaP abl cells were treated with 30 µM bicalutamide (Selleck Chemicals, Houston, TX, USA) for 7 days. LNCaP, LNCaP abl and PC3 cells were treated with 17β-oestradiol (Sigma) at a concentration of 10 nM. Bicalutamide and 17β-oestradiol were dissolved in dimethyl sulfoxide (DMSO).

### Bioinformatics analysis

The Best.C (GSE2443) [22], Goodison.S (GSE25136) [23], Monzon (GSE6919) [24, 25], and Nelson (GSE74367) [26] Gene Expression Omnibus (GEO) datasets (http://www.ncbi.nlm.nih.gov/geo/) from PubMed were used in our study. For correlation analysis of ERα and NRF2 expression, we used mRNA expression data from human prostate cancer datasets from The Cancer Genome Atlas (TCGA), including data for 498 prostate tumour tissues and 52 adjacent normal tissues, and Genotype-Tissue Expression (GTEx), including data for 100 normal prostate tissues. Gene set enrichment analysis (GSEA) was performed using the GSEA software package (GSEA-3.0.jar) [27]. Clinical outcome analyses were performed using GraphPad Prism 5.

### Generation of PCa cell lines stably expressing NRF2 short hairpin RNA

The lentiviral shuttle plasmid and its original packaging and envelope plasmids were prepared (psPAX2, pMD2.G and pHBLV). An endotoxin-free plasmid extraction kit was used for plasmid extraction, and 293T cells were then transfected with 5 μg of NRF2 short hairpin RNA (shNRF2; the sequence is shown in Additional file 2: Table S1), the psPAX2 plasmid, and the pMD2.G plasmid; 5 μg of the pHBLV plasmid was used as a control. Culture supernatant containing lentiviral particles was collected at 48 and 72 h. Half of the final concentration of puromycin needed to kill control cells was used to culture virus-infected cells.

### Small interfering RNA and transient transfection

Control small interfering RNA (siRNA) and siRNAs targeting ERα, ERβ, G protein-coupled receptor 30 (GPR30), AR, and NRF2 were purchased from GenePharma (Shanghai, China). The siRNA sequences are listed in Additional file 2: Table S2. All transfections with siRNA and plasmids were conducted using Lipofectamine 3000 (Invitrogen) following the manufacturer’s instructions.

### Chromatin immunoprecipitation

PC3 cells were treated with DMSO or 10 nM 17β-oestradiol for 30 min before harvesting. Chromatin immunoprecipitation (ChIP) assays were performed as previously described [28]. DNA was sonicated, purified, extracted, and analysed by PCR. The primers used in the ChIP assays are shown in Additional file 2: Table S2, and an anti-ERα antibody (ab32063; Abcam, Cambridge, MA, USA) was used.

### Western blot analysis and quantitative PCR

Total RNA was extracted from PCa cells with TRIzol reagent (Invitrogen) according to the manufacturer’s instructions and was then reverse transcribed into cDNA. Quantitative PCR (qPCR) was performed as previously described [29]. The housekeeping gene in PCa cells is hypoxanthine phosphoribosyl transferase 1. The primer sequences are shown in Additional file 2: Table S2. Protein lysis buffer (RIPA; Thermo Fisher, Waltham, MA, USA) was used to extract total protein from cells, and Western blot analysis was performed as previously described [30]. The primary antibodies used are listed in Additional file 2: Table S3.

### Immunohistochemical and immunofluorescence analyses

Prostate tissue samples from CRPC patients (age 63–78 years, n = 28) who underwent surgical castration and primary PCa tissues from patients who underwent radical prostatectomy (age 63–78 years, n = 34) were obtained from the Department of Pathology and the Department of Urology, Second Hospital of Tianjin Medical University (Tianjin, China). Normal human prostate specimens from patients undergoing radical cystectomy for bladder cancer (age 53–67 years, n = 21) were obtained from the Department of Urology, Shanghai First People’s Hospital (Shanghai, China). All samples were obtained with the informed consent of the patients, and approval of the study was obtained from the Ethics Committee of the Second Hospital of Tianjin Medical University (code: 2020KYLL081102, Tianjin, China). Immunohistochemical (IHC) and immunofluorescence (IF) analyses were performed on prostate tissues as previously described [31]. The primary antibodies are provided in Additional file 2: Table S3. For IF analysis, LNCaP and LNCaP abl cells were treated with bicalutamide or 10 nM E2 for 72 h, and IF images were acquired using a fluorescence microscope (Leica, Wetzlar, Germany). IHC images were acquired using an Olympus microscope (BX43; Olympus, Tokyo, Japan).

### Luciferase reporter assay

The NRF2 promoter fragments with the wild-type or mutated ERE were synthesized by and purchased from Shanghai General Biology (Anhui), and the pGL3-Basic vector (Promega, Madison, WI, USA) was used to construct the Normal-luc and Mutation-luc plasmids. The plasmids were transfected into PCa cells after the indicated treatment for 24 and 48 h. The sequences of the promoter fragments in the Normal-luc and Mutation-luc plasmids are shown in Additional file 2: Table S4. Luciferase activity was measured using a dual-luciferase assay kit (Promega, Madison, WI, USA) as previously described [32].

### Cell proliferation and migration assays

To monitor proliferation, cells were transfected with shRNAs or ERα and NRF2 overexpression plasmids for 24 h prior to bicalutamide treatment (0, 10, 20, 30, 40 and 50 μM). After 48 h, the sensitivity of the different treatment groups to bicalutamide was determined by an MTT assay (with the absorbance measured at 570 nm). A scratch assay was used to detect the migration of PC3 cells, which were divided into different treatment groups. The cells were cultured in a 6-well plate and scratched with the fine end of a 1 mL pipette tip. Images were acquired at 0 and 24 h. In addition, Transwell assays were used to evaluate the migration ability of PC3 cells. Briefly, cells suspended in RPMI 1640 medium were seeded in Transwell migration chambers (Transparent PET Membrane 24-well, 8.0 µm pore size; Corning Inc., Corning, NY, USA), and RPMI 1640 medium supplemented with 10% FBS was added to the lower compartments. The cells that migrated through the filter to the lower surface of the membrane were fixed with 4% paraformaldehyde and stained with a 0.5% crystal violet solution (BBI Life Sciences, Shanghai, China) after overnight incubation. Finally, the cells were counted in five randomly selected areas in each chamber.

### Statistical analyses

Data are shown as the mean ± standard error of the mean (SEM) values. Student's *t* test was used to analyse differences between two groups, and differences among more than two groups were compared with one-way ANOVA. *P* < 0.05 was considered significant.

## Results

### NRF2 and ERα are highly expressed and positively correlated in human CRPC tissues and cell lines

**Fig. 1:**
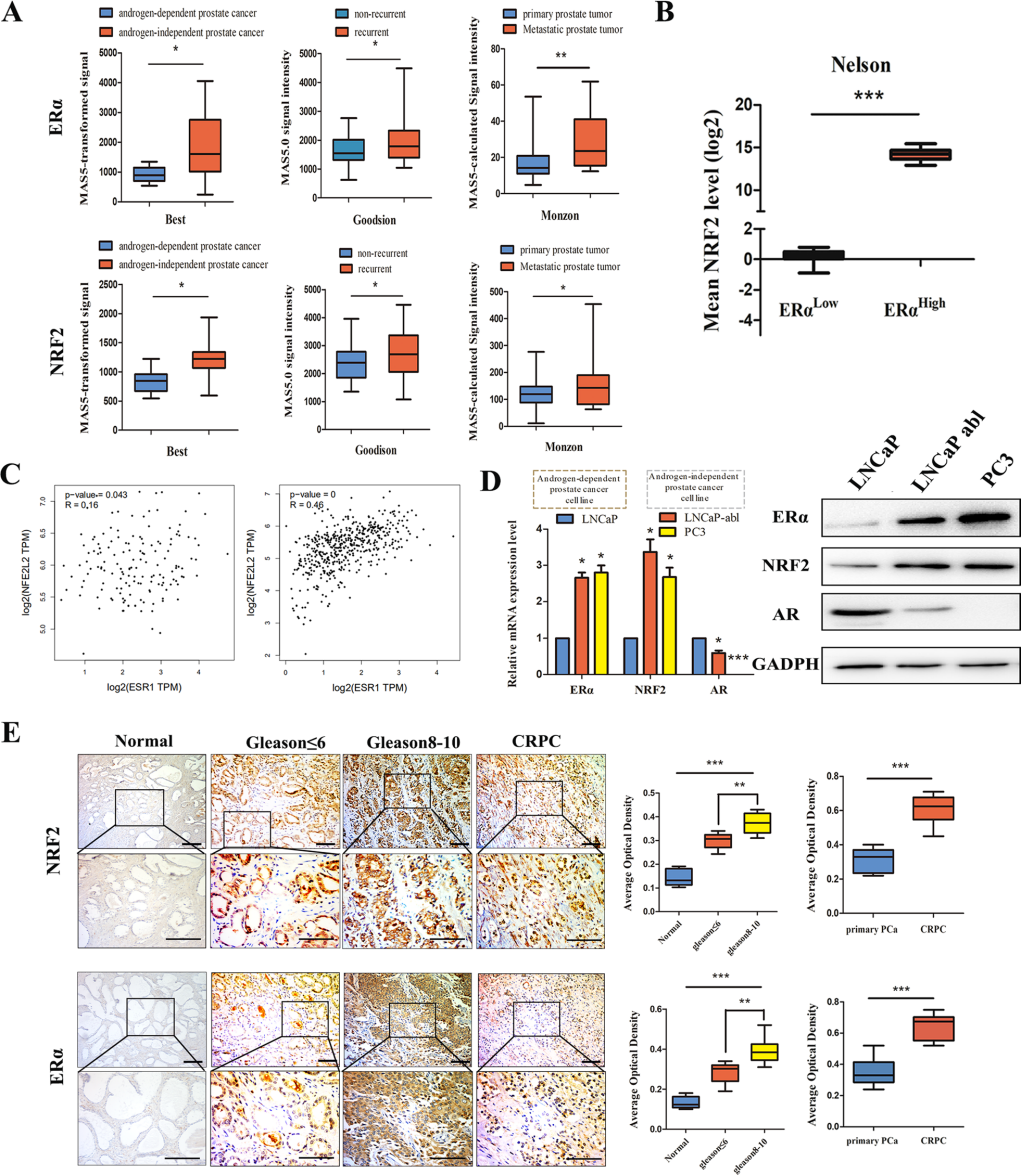
Expression of ERα and NRF2 in PCa. **A** Box and whisker plots showing ERα and NRF2 mRNA expression in different PCa tissues from three publicly available datasets (*t* test). **B** Analysis of mean NRF2 mRNA expression in ERα^High^ samples vs. ERα^Low^ samples in prostate adenocarcinoma (PRAD) data from the Nelson dataset (*t* test). **C** The correlation of ERα and NRF2 expression in normal prostate and PRAD patients from TCGA (n (Normal) = 52), n (PRAD) = 498)) and GTEx (n (Normal) = 100) datasets. **D** The mRNA levels of ERα, NRF2 and AR in LNCaP, LNCaP abl, and PC3 cells are shown (one-way ANOVA). **E** Immunohistochemical analysis of ERα and NRF2 in normal prostate tissues (n = 21), PCa tumours with a low Gleason score (Gleason ≤ 6) (n = 17), PCa tumours with a high Gleason score (Gleason 8–10) (n = 17) and CRPC tissues (n = 28); representative microphotographs are shown; scale bar, 100 μm. The right panel shows the average optical density of ERα and NRF2 in normal prostate vs. low Gleason score vs. high Gleason score tissues and primary tumour vs. CRPC tissues (*t* test). The average optical density was measured with Image-Pro Plus 6.0 software; the values are presented as the means ± SEMs; *P < 0.05, **P < 0.01, ***P < 0.001

We examined the expression of NRF2 and ERα in different PCa tissues from three publicly available datasets (Best.C, 10 androgen-dependent PCa and 10 androgen-independent PCa samples; Goodison.S, 40 nonrecurrent primary PCa and 39 recurrent primary PCa samples; Monzon, 64 primary PCa and 25 metastatic PCa samples). NRF2 expression was significantly higher in CRPC than in primary PCa (Fig. 1A). We grouped ERα^High^ and ERα^Low^ PCa tumour samples from a GEO dataset (Nelson, 11 primary PCa tumour and 45 CRPC tumour samples). The mean NRF2 gene expression level was much higher in ERα^High^ prostate adenocarcinoma samples than in ERα^L^°^w^ prostate adenocarcinoma samples (Fig. 1B). Thus, we focused on the relationship between ERα and NRF2 expression. We analysed the correlation of ERα and NRF2 expression in the TCGA and GTEx projects. The results showed that the correlation between ERα and NRF2 expression was more significant in PCa than in normal prostate tissue (Fig. 1C). Next, we performed quantitative PCR and Western blotting to assess the mRNA and protein expression, respectively, of ERα and NRF2 in PCa cell lines. ERα and NRF2 expression was significantly upregulated in the androgen-independent PCa cell lines (LNCaP abl and PC3) compared with the androgen-dependent PCa cell line (LNCaP). AR had the opposite expression pattern to ERα and NRF2 (Fig. 1D). In addition, IHC staining was performed to evaluate the protein expression of ERα and NRF2 in normal prostate (n = 21), primary PCa (n = 34), and CRPC (n = 28) samples. ERα and NRF2 were both significantly upregulated in prostate tumours compared with normal tissues. In the primary PCa samples, tumours with a high Gleason score (Gleason 8–10) had stronger ERα and NRF2 staining than those with a low Gleason score (Gleason ≤ 6). The CRPC samples exhibited higher ERα and NRF2 expression than the primary PCa samples (Fig. 1E). Our results demonstrate that ERα and NRF2 expression are correlated and are associated with prostate cancer progression.

### NRF2 expression is enhanced in association with ERα upregulation upon bicalutamide treatment

**Fig. 2:**
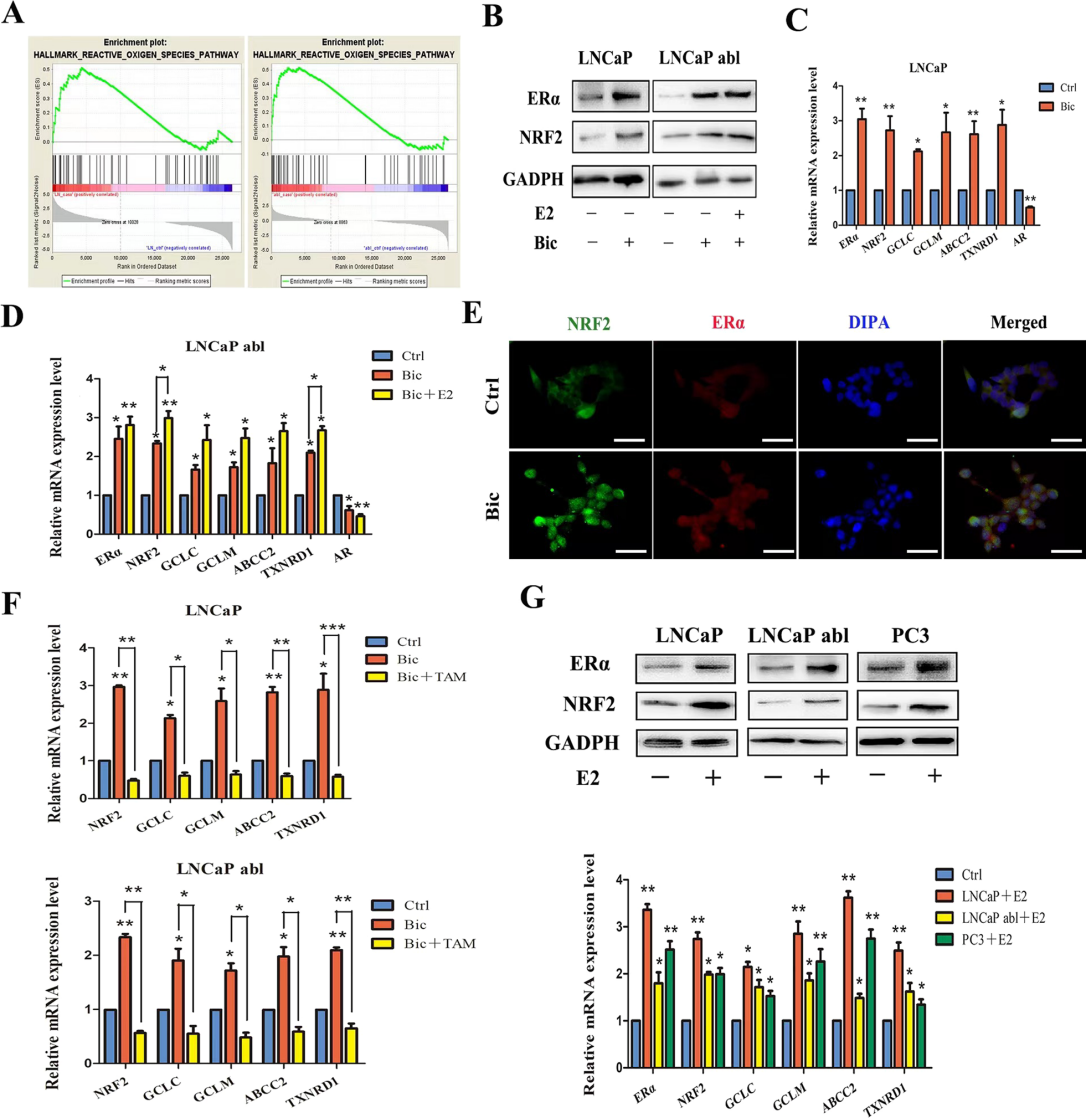
NRF2 expression was enhanced in association with ERα upregulation upon bicalutamide treatment in PCa cells. LNCaP and LNCaP abl cells were treated with 30 μM bicalutamide for 7 days, and LNCaP abl cells were exposed to 10 nM 17β-oestradiol (E2). **A** GSEA plot of ROS pathway gene set activity in PCa cells from mRNA-seq data. **B** The protein levels of ERα and NRF2 were analysed. **C**, **D** The mRNA levels of ERα, NRF2, AR, and NRF2 target genes were analysed in LNCaP (*t* test) and LNCaP abl (one-way ANOVA) cells. **E** Representative graphs of ERα and NRF2 immunofluorescence data in LNCaP cells before and after bicalutamide treatment; scale bar, 50 μm. **F** mRNA levels of ERα, NRF2, and its target genes in LNCaP cells and LNCaP abl cells exposed to bicalutamide and tamoxifen (one-way ANOVA). **G** mRNA and protein levels of ERα, NRF2 and NRF2 target genes in LNCaP and LNCaP abl cells treated with DMSO or 10 nM E2 (*t* test). All studies were repeated at least three times; the values are presented as the means ± SEMs; *P < 0.05, **P < 0.01, ***P < 0.001

To identify the role of NRF2 pathway activation in the progression of bicalutamide-resistant PCa, we treated LNCaP and LNCaP abl cells with an AR blocker, bicalutamide. After 7 days of exposure to 30 μM bicalutamide, we conducted GSEA on the mRNA-seq dataset. The reactive oxygen species (ROS) pathway was enriched in genes upregulated after treatment with bicalutamide in LNCaP and LNCaP abl cells (Fig. 2A). At the cellular level, we detected increases in ERα and NRF2 protein expression in LNCaP and LNCaP abl cells treated with bicalutamide (Fig. 2B). At the mRNA level, increased expression of ERα and NRF2, along with NRF2 target genes, and lower expression of AR were observed in LNCaP and LNCaP abl cells treated with bicalutamide (Fig. 2C, D). To explore the potential effect of oestrogen on the acquisition of bicalutamide resistance, we treated LNCaP abl cells with 10 nM 17β-oestradiol in combination with bicalutamide and investigated the expression of ERα and NRF2. Combined E2+bicalutamide treatment upregulated the expression of NRF2 compared to bicalutamide treatment alone. Moreover, IF was conducted to investigate whether NRF2 and ERa are expressed in the same cell. As expected, ERα and NRF2 were coexpressed in LNCaP cells after bicalutamide treatment (Fig. 2E). Thus, we hypothesized that the effects of oestrogen promote NRF2 signalling in PCa cells. Treatment with 100 nM tamoxifen (TAM) blocked the upregulation of ERα and NRF2 expression induced by bicalutamide (Fig. 2F). This indicated that NRF2 is regulated in an ERα-mediated oestrogen-dependent manner. To determine whether E2 regulates NRF2 expression, we detected the expression of NRF2 in LNCaP, LNCaP abl, and PC3 cells treated with E2. The results showed that E2 promoted the expression of NRF2 and its target genes (Fig. 2G). IF analysis confirmed the above conclusion in LNCaP abl cells (Additional file 3: Fig. S1A). We established PC3 and LNCaP abl cell lines with stable knockdown of NRF2, and Western blotting was used to determine the knockdown efficiency (Additional file 3: Fig. S1B). The changes in gene expression induced by E2 were inhibited by NRF2 knockdown (Additional file 3: Fig. S1C).

### ERα binds directly to the ERE in the NRF2 promoter

**Fig. 3:**
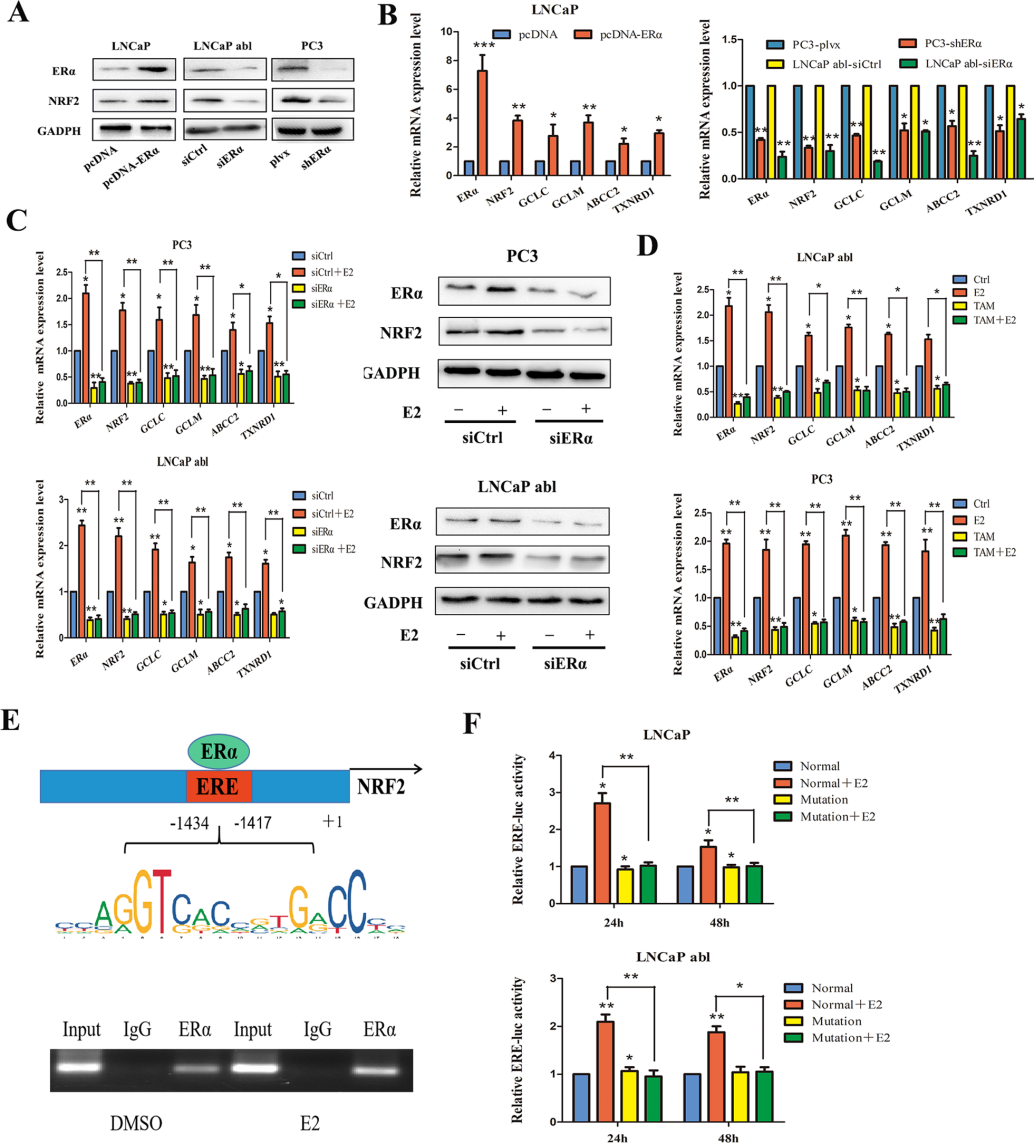
The mechanisms by which NRF2 is regulated by E2. **A** Western blot analysis of the expression of NRF2 in LNCaP abl and PC3 cells with ERα knockdown and in LNCaP cells with ERα overexpression. **B** qPCR analysis of the expression of NRF2 and its target genes (*t* test). **C** qPCR and Western blot analysis of the indicated molecules in LNCaP abl or PC3 cells with or without ERα knockdown after treatment with either DMSO or 10 nM E2 (one-way ANOVA). **D** qPCR analysis of the indicated molecules in LNCaP abl or PC3 cells treated with or without TAM after treatment with either DMSO or 10 nM E2 (one-way ANOVA). **E** ChIP assay showing that ERα binds directly to the ERE in the NRF2 promoter region. **F** ERα transcriptional activity in LNCaP and LNCaP abl cells after different treatments (one-way ANOVA). All studies were repeated at least three times; the values are presented as the means ± SEMs; *P < 0.05, **P < 0.01, ***P < 0.001

To explore the molecular mechanisms by which E2 regulates NRF2, ERα was knocked down in LNCaP abl and PC3 cells and overexpressed in LNCaP cells. The results showed that the expression of NRF2 was downregulated in LNCaP abl and PC3 cells with ERα knockdown and that overexpression of ERα in LNCaP cells significantly increased the protein expression of NRF2 (Fig. 3A). Furthermore, overexpression of ERα in LNCaP cells increased the mRNA expression of NRF2 and its target genes glutamate-cysteine ligase catalytic subunit (GCLC), glutamate-cysteine ligase modifier subunit (GCLM), ATP binding cassette subfamily C member 2 (ABCC2) and thioredoxin reductase 1 (TXNRD1), while ERα knockdown in LNCaP abl and PC3 cells decreased the expression of these genes (Fig. 3B). Next, we knocked down ERα in LNCaP abl and PC3 cells before treatment with or without E2. ERα knockdown blocked the E2-induced upregulation of ERα, NRF2 and its target genes (Fig. 3C). The ERα inhibitors TAM and ICI182780 exerted a similar effect on these genes during E2 treatment (Fig. 3D, Additional file 3: Fig. S2A). Next, we analysed the NRF2 promoter for the presence of an ERE, which facilitates the direct binding of ERα to the promoter sequence. Indeed, we found a putative ERE in the NRF2 promoter, which was further confirmed by the ChIP assay. Our findings suggested that E2 stimulated NRF2 transcription by inducing the binding of ERα to a specific ERE in the NRF2 promoter region (Fig. 3E). To test this hypothesis, we used the Dual-Luciferase ® Reporter Assay System to measure the activity of the normal ERE (Normal) and mutated ERE (Mutation) in LNCaP and LNCaP abl cells treated with or without E2. In LNCaP and LNCaP abl cells, the NRF2 promoter fragment with the normal ERE sequence exhibited increased transcriptional activity after treatment with E2 for 24 and 48 h. On the other hand, the activity of the NRF2 promoter with the mutated ERE sequence did not change (Fig. 3F). Finally, to determine whether AR regulates the expression of NRF2, the results showed that knocking down AR in LNCaP, LNCaP abl, and PC3 cells had no effect on the expression of NRF2 (Additional file 3: Fig. S2B). To rule out the possibility that other oestrogen receptors also regulate NRF2, we individually knocked down ERα, ERβ and GPR30 in LNCaP abl and PC3 cells. Only knockdown of ERα decreased NRF2 expression, indicating that ERα regulates NRF2 expression (Additional file 3: Fig. S2C).

### Inhibition of the ERα-induced NRF2 signalling pathway resensitizes bicalutamide-resistant cells to bicalutamide

**Fig. 4:**
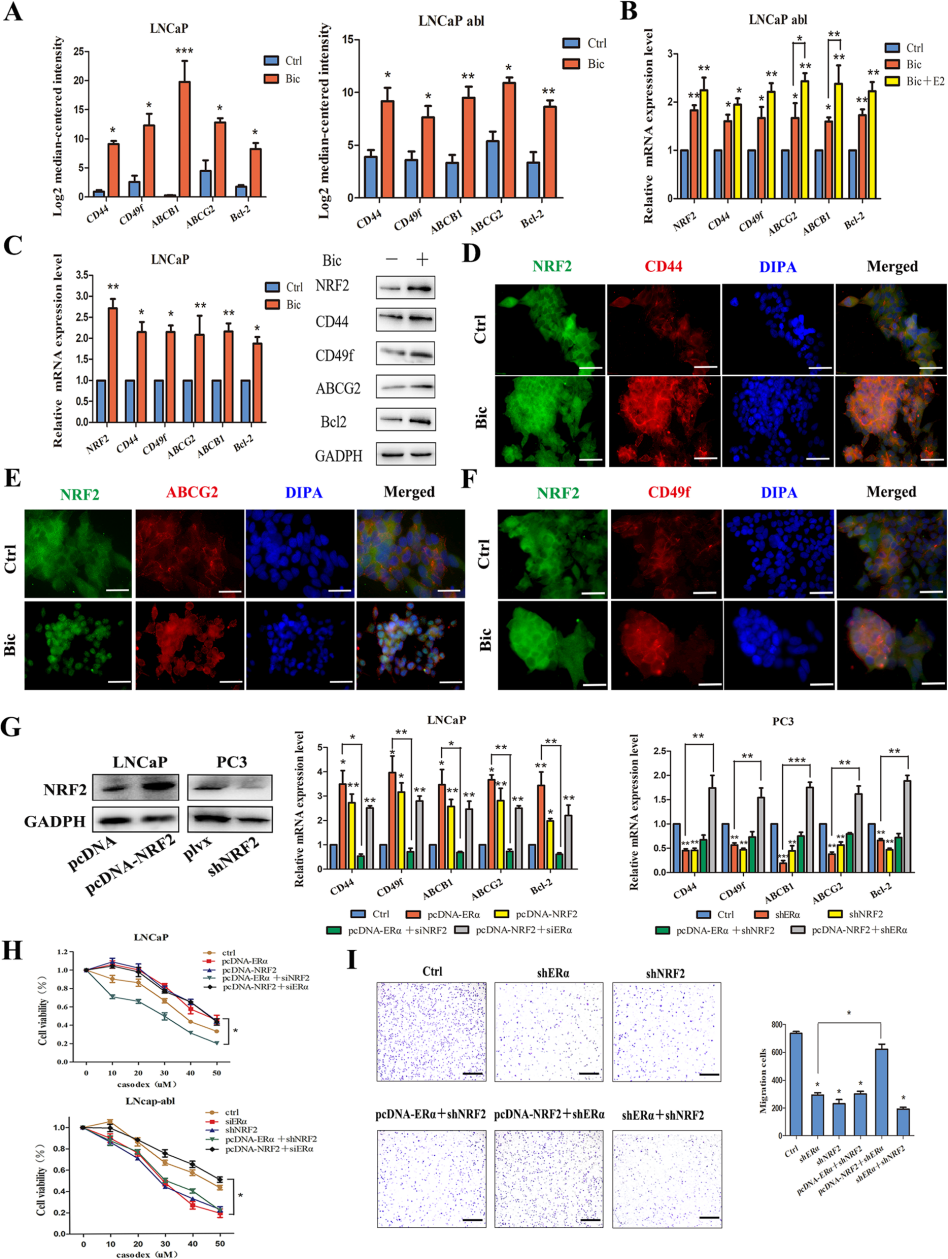
The ERα-NRF2 signalling axis enhances bicalutamide resistance. **A** mRNA-seq of LNCaP and LNCaP abl cells treated with DMSO or 30 μM bicalutamide (*t* test). **B**, **C** The mRNA and protein levels of bicalutamide resistance-related genes were analysed in LNCaP cells (*t* test) and LNCaP abl cells (one-way ANOVA). **D**–**F** Representative graphs of IF data for NRF2 coexpression with CD44 and ABCG2 in LNCaP cells. Scale bar, 50 μm. **G** qRT‒PCR analysis showing expression changes in resistance-related genes in LNCaP abl cells with ERα knockdown and LNCaP cells with ERα overexpression subjected to different treatments (one-way ANOVA). **H** MTT assay of LNCaP and LNCaP abl cells (*t* test) after treatment with 30 μM bicalutamide. **I** Invasion ability was assessed in PC3 cells subjected to different treatments (one-way ANOVA). Scale bar, 500 μm. All studies were repeated at least three times; the values are presented as the means ± SEMs; *P < 0.05, **P < 0.01, ***P < 0.001

**Fig. 5:**
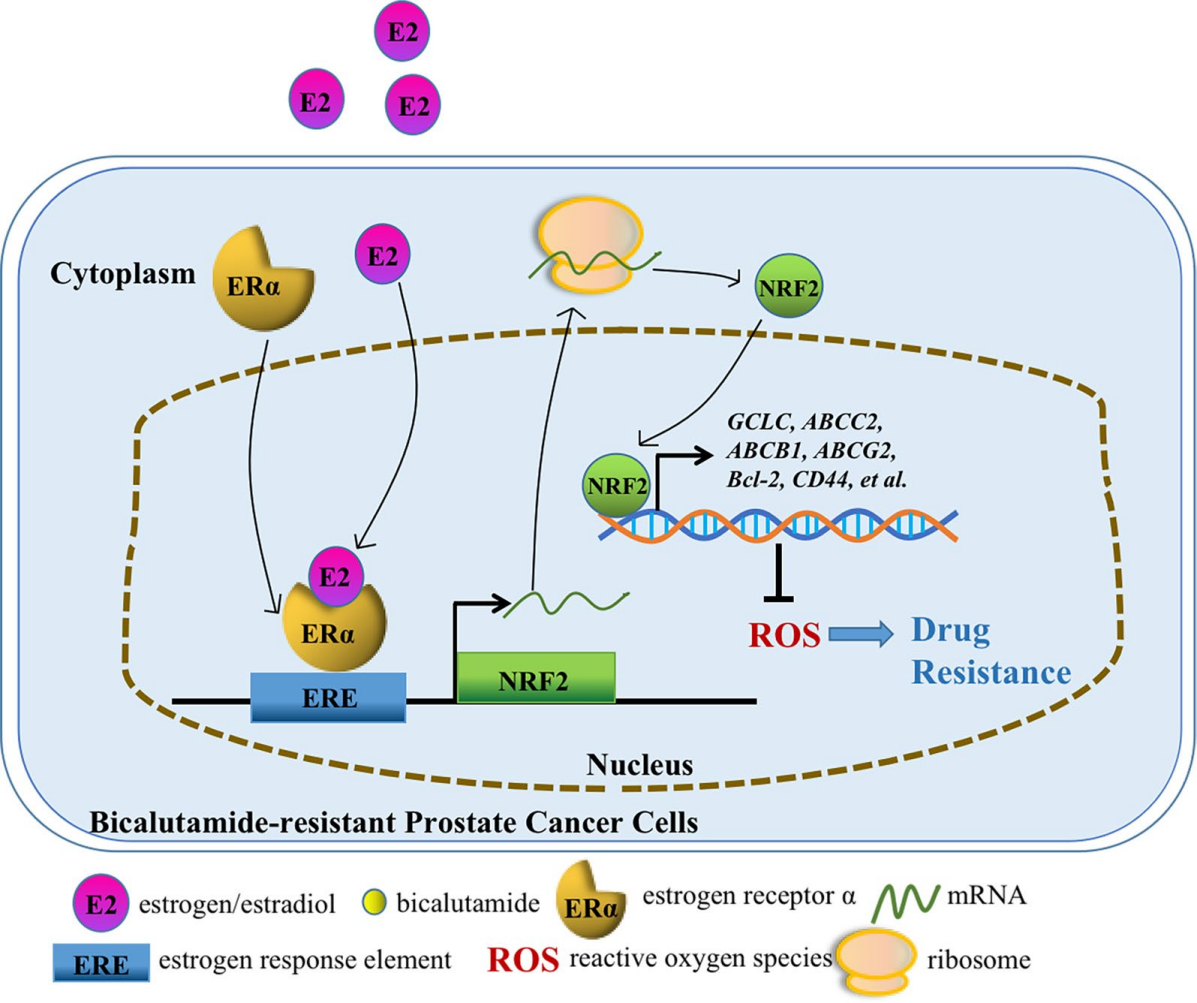
The overall signalling pathway. ERα-induced effects of oestrogen promote the expression of NRF2 by the binding of ERα to the NRF2 promoter at the site between − 1434 and − 1417 bp. The ERα-NRF2 signalling axis enhances the sensitivity of prostate cancer cells to bicalutamide by promoting the expression of resistance-related genes

To explore the potential mechanism inducing resistance to bicalutamide in prostate cancer cells, we treated LNCaP and LNCaP abl cells with bicalutamide and collected surviving cells after 7 days for mRNA analysis. We found that the expression of the antiapoptotic gene BCL2, genes encoding ATP-binding cassette transporters such as ABCG2 and ABCB1, and the stemness genes CD44 and CD49f was upregulated in surviving LNCaP and LNCaP abl cells (Fig. 4A). The above genes may promote resistance to bicalutamide and can be defined as bicalutamide resistance-related genes. Then, qPCR and Western blot analysis verified that treatment with bicalutamide upregulated the expression of resistance-related genes in LNCaP and LNCaP abl cells (Fig. 4B, C). IF analysis was performed to analyse the increased expression and the coexpression of NRF2 and CD44/ABCG2/CD49f in LNCaP cells after bicalutamide treatment (Fig. 4D–F). Furthermore, qPCR analysis was performed, and the results indicated that NRF2 silencing abolished the ERα-induced upregulation of resistance-related genes in LNCaP and PC3 cells. Overexpression of NRF2 rescued the expression of resistance-related genes in LNCaP and PC3 cells with ERα silencing (Fig. 4G). These results suggested that the ERα-NRF2 signalling axis may play an important role in the acquisition of resistance to bicalutamide by inducing the expression of resistance-related genes. To determine whether ERα–NRF2 signalling regulates bicalutamide resistance in PCa cells, shRNAs targeting NRF2 and ERα were used to silence the expression of NRF2 and ERα. The MTT assay showed that ERα overexpression did not regulate the sensitivity of LNCaP abl cells to bicalutamide when NRF2 was silenced. However, NRF2 overexpression rescued the viability of LNCaP cells upon ERα knockdown (Fig. 4H). In addition, knockdown of ERα and NRF2 decreased PC3 cell migration. Overexpression of NRF2 rescued the migration of PC3 cells with ERα knockdown, but overexpression of ERα in cells with NRF2 knockdown had no effect (Fig. 4I). The scratch assay showed similar results (Additional file 3: Fig. S3). Furthermore, we analysed the antiapoptotic and proapoptotic genes expression in LNCaP and LNCaP abl cells with or without bicalutamide treatment for 7 days via mRNA-seq assays. And the results showed that the antiapoptotic genes BCL2, BCL2L1 in LNCaP abl cells and BCL2, BCL2L2 in LNCaP cells were up-regulated with bicalutamide treatment and their expressions were positively correlated with the expression of NRF2, while the proapoptotic gene BID was downregulated in LNCaP cells with bicalutamide treatment (Additional file 3: Fig S4). These results indicate that the ERα–NRF2 signalling axis is beneficial for bicalutamide resistance in prostate cancer cells (Fig. 5).

## Discussion

Drug resistance remains a major obstacle in the treatment of PCa. Generally, several factors have been shown to be involved in the mechanisms of bicalutamide resistance in PCa [33–35]. We propose that the expression of aromatase is increased upon bicalutamide treatment and that the catalysis of endogenous oestrogen biosynthesis from testosterone is increased. Mechanistically, aromatase-induced oestrogen promotes the binding of ERα to the ERE in the matrix metalloproteinase 12 promoter upon bicalutamide treatment in PCa cells, leading to CRPC progression [11]. This study further explains the molecular mechanisms underlying the effects of oestrogen on promoting the acquisition of bicalutamide resistance in PCa cells.

As previously described, oestrogen exerts its biological effects on target tissues mainly by binding to specific intracellular receptors, ERα and ERβ [36–38]. However, recent studies have provided evidence that luminal cells with high ERβ expression are particularly fragile and undergo programmed cell death after ADT, radiation, and chemotherapy. Basal cells that express ERα are multidrug-resistant and can survive in cytotoxic conditions [10]. Previous studies indicated that NRF2 plays a role in drug resistance in a variety of cancers, such as breast cancer, hepatocellular carcinoma, bladder cancer, and pancreatic cancer [39]. Oxidative stress promotes the initiation, progression, and transition of PCa to CRPC [40–42], and NRF2, the transcription factor that plays a key role in the response to oxidative stress, significantly decreases transactivation of AR and suppresses dihydrotestosterone-induced AR activity [13, 43]. However, the regulatory effects of oestrogen on NRF2 expression are poorly understood in PCa cells resistant to bicalutamide. In this study, by analysing PCa sample data obtained from the GEO and TCGA databases, we found that the expression of NRF2 was significantly upregulated in androgen-independent PCa cells and CRPC tissues and was significantly positively correlated with ERα expression. The expression of both ERα and NRF2 was significantly increased, and these proteins were coexpressed and colocalized after treatment with bicalutamide. Additionally, inhibiting the expression of ERα not only reduced NRF2 expression but also blocked the upregulation of NRF2 induced by E2. Based on these findings, we hypothesized that the ERα-NRF2 signalling axis is a potential pathway contributing to bicalutamide resistance in PCa cells. As expected, overexpression of NRF2 desensitized PCa cells to bicalutamide and rescued the cell migration inhibited by ERα knockdown. The expression of antiapoptotic genes was upregulated in bicalutamide resistant cells and positively correlated with NRF2 expression. Whether NRF2 signaling pathway induces bicalutamide resistance through cell escape from bicalutamide-induced apoptosis remains to be studied. Moreover, NRF2 knockdown reduced the expression of detoxifying enzymes induced by E2, indicating that the effect of oestrogen mediates bicalutamide resistance by regulating reactive oxygen species homoeostasis, suggesting a critical role of the ERα-NRF2 signalling axis in regulating bicalutamide resistance in PCa cell lines and providing a potential diagnostic and predictive biomarker for PCa.

We further investigated the regulatory mechanism of ERα and NRF2 expression. The ERα antagonist MPP inhibits NRF2 activity in MCF-7 breast cancer cells, and oestrogen increases NRF2 activity in MCF7 breast cancer cells by activating the phosphoinositide 3-kinase/glycogen synthase kinase 3 beta (PI3K/GSK3β) pathway [16]. Our results showed that E2 regulates NRF2 via the binding of ERα to the ERE in the promoter of NRF2 upon bicalutamide treatment in PCa cells, indicating that NRF2 is a direct target gene of ERα and mediates bicalutamide resistance in CRPC as an ERα-mediated downstream effect of oestrogen. However, oestrogen has been shown to recruit ERα to the NQO1 promoter, resulting in inhibited transcription of NQO1, an NRF2-dependent detoxification enzyme, in turn contributing to breast cancer prevention [44]. Further study is needed to understand the regulatory relationship between ERα and NRF2 in different cancer types.

In several types of cancers, CD44 was consequently recognized as a cancer stem cell marker that has been indicated to participate in tumour progression, metastasis and drug resistance [45–47]. Previous studies suggested that PCa stem-like cells are a key contributor to CRPC. The population of CD44^+^ stem-like cells is increased with PCa progression after ADT, and aromatase is expressed mostly in CD44^+^ PCa cells [23]. Our data showed that the ERα-induced effects of oestrogen increased the expression of CD44 by regulating NRF2, while NRF2 knockdown reduced CD44 expression and increased sensitivity to bicalutamide. Moreover, CD44 and NRF2 showed coexpression in CRPC tissue after bicalutamide treatment. Therefore, NRF2 might promote bicalutamide resistance in CRPC cells by regulating the expression of CD44. However, Ryoo et al. [48] reported that the molecular mechanisms of CD44-mediated NRF2 activation involve high p62 expression and that high levels of CD44 lead to p62-associated NRF2 activation in CD44^high^ breast cancer stem cell (CSC)-like cells. Therefore, future studies are needed to determine the role of the interaction between CD44 and NRF2 in CRPC.

In vivo experiments can be conducted to further verify the mechanism of ERα-mediated NRF2 expression in bicalutamide resistance. Future studies are also needed to determine whether there is a molecular mechanism similar to that for bicalutamide resistance mediating resistance to other androgen receptor antagonists in PCa.

## Conclusions

This research showed the coexpression and colocalization of ERα and NRF2 in CRPC tissues and cell lines and demonstrated that ERα directly binds to the ERE in the NRF2 promoter to increase the expression of NRF2 in PCa cells treated with bicalutamide. Moreover, we verified that activation of the ERα-NRF2 signalling pathway contributes to bicalutamide resistance in PCa cells. These results suggest that the ERα-NRF2 signalling pathway is a potential therapeutic target for PCa.

## Supplementary Information

**Additional file 1.** Supplementary description of cell lines. In this additional file, the characteristics and culture methods of bicalutamide sensitive cells and resistant cells, as well androgen-dependent and independent cells were detailly described.

**Additional file 2. Supplementary Tables. Table S1:** shNRF2 sequences. **Table S2:** Primer sequences. **Table S3:** List of antibodies. **Table S4:** Plasmids sequences.

**Additional file 3. Supplementary Figures. Fig. S1.** (**A**) Representative IF graphs of NRF2 in LNCaP abl cells treated with DMSO or 10 nM E2 treatment; scale bar, 50 μm. (**B**) Western blot analysis showing LNCaP abl or PC3 with NRF2 knockdown. (**C**) qPCR analysis showing expression changes of the indicated genes in LNCaP abl and PC3 treated with either DMSO or 10 nM E2 (one-way ANOVA). All studies were repeated at least three times. The data are presented as the mean ± SEM. * P < 0.05, **P < 0.01. **Fig. S2.** (**A**) qPCR analysis of the indicated in LNCaP abl or PC3 cells with or without ICI182780 after treatment with either DMSO or 10 nM E2 (one-way ANOVA). (**B**) qPCR and Western blot analysis of LNCaP, LNCaP abl, and PC3 cells transfected with siAR and siCtrl. qPCR analysis showing expression changes of NRF2(t-test). (**C**) qPCR and Western blot analysis were conducted in LNCaP abl and PC3 cells transfected with siERα, siERβ, siGPR30 and siCtrl. qPCR analysis showing expression changes of NRF2(t-test). All studies were repeated at least three times; the values are the mean ± SEM; *P < 0.05, **P < 0.01. **Fig. S3.** Scratch test was conducted in PC3 cells with different treatments, scale bar, 200 μm. All studies were repeated at least three times; the values are the mean ± SEM; *P < 0.05. **Fig. S4.** The mRNA-seq analysis indicated that the expression of antiapoptotic genes was up-regulated in bicalutamide resistant cells and positively correlated with the expression of NRF2. The heatmap was generated by an online analytic tool (www.xiantao.love).

## Data Availability

The data and materials are availability from corresponding authors on reasonable request.

## Abbreviations

ABCB1: ATP binding cassette subfamily B member 1
ABCC2: ATP binding cassette subfamily C member 2
ABCG2: ATP binding cassette subfamily G member 2
ADT: Androgen deprivation therapy
AR: Androgen receptor
ChIP: Chromatin immunoprecipitation
CRPC: Castration-resistant prostate cancer
DMSO: Dimethyl sulfoxide
E2: 17β-Estradiol
ERα: Estrogen receptor α
ERβ: Estrogen receptor β
ERE: Estrogen response element
FBS: Fetal bovine serum
GCLC: Glutamate-cysteine ligase catalytic subunit
GCLM: Glutamate-cysteine ligase modifier subunit
GEO: Gene expression omnibus
GPR30: G protein-coupled receptor 30
GSEA: Gene set enrichment analysis
GTEx: Genotype-tumor expression
IF: Immunofluorescence
IHC: Immunohistochemistry
NGR1: Notoginsenoside R1
NRF2: Nuclear factor E2-related factor 2
PCa: Prostate cancer
ROS: Reactive oxygen species
TAM: Tamoxifen
TCGA: The Cancer Genome Atlas
TXNRD1: Thioredoxin reductase 1
