# Supplementary material for: The ERα-NRF2 signalling axis promotes bicalutamide resistance in prostate cancer
Source: Cell Commun Signal. 2022 Nov 14;20:178. doi: 10.1186/s12964-022-00979-0 (PMC9661764; doi:10.1186/s12964-022-00979-0)
Supplement: Supplementary file 2 — Additional file 1. Supplementary description of cell lines. In this additional file, the characteristics and culture methods of bicalutamide sensitive cells and resistant cells, as well androgen-dependent and independent cells were detailly described. [file 12964_2022_979_MOESM2_ESM.docx]

**Supplementary description of cell lines**:

**1**. **Bicalutamide sensitive cells and resistant cells**. To generate bicalutamide resistant PCa cells, LNCaP and LNCaP abl cells were treated with 30 μM bicalutamide for 1 week, and the surviving cells exhibited bicalutamide resistance according to our previous report (Lin et al., Front Oncol, 2022). The expression of bicalutamide resistance related genes, such as ABCG2, ABCB1, BCL2, CD44 and CD49f, cell proliferation and migration were detected to confirm bicalutamide resistance of PCa cells.

**2.** **Androgen-dependent and androgen-independent cells**. LNCaP cells positively express androgen receptor (AR) and the 5-α-dihydrotestosterone can promote their growth. The cells are widely recognized as androgen-dependent cells in previous reports (NickKholgh B et al., Prostate, 2016; Singh VK et al., Environ Pollut, 2021). While, the LNCaP abl cells were established from LNCaP cells which were kept in androgen-depleted medium for 87 passages (Z Culig et al., Br J Cancer, 1999). And the cells are cultured in androgen-free medium and its growth is not dependent on androgen. Thus, LNCaP abl cells are androgen-independent. The PC3 cells negatively express AR and are recognized as androgen-independent cells (Chen Q, Int J Mol Sci, 2016).

**3.** **Culture methods**. The LNCaP and PC3 cells were cultured in RPMI1640 medium supplemented with 1% penicillin/streptomycin (P/S) and 10% foetal bovine serum (FBS). Considering that normal FBS contains certain levels of steroid hormones including androgen, the charcoal dextran stripped foetal bovine serum (CDS-FBS), in which the steroid hormones are effectively removed, instead of FBS was used to culture LNCaP abl cells to maintain its androgen-independent characteristics which was consistent with the culture method described in the previous literature (Z Culig et al., Br J Cancer, 1999).
