# Supplementary material for: The ERα-NRF2 signalling axis promotes bicalutamide resistance in prostate cancer
Source: Cell Commun Signal. 2022 Nov 14;20:178. doi: 10.1186/s12964-022-00979-0 (PMC9661764; doi:10.1186/s12964-022-00979-0)
Supplement: Supplementary file 3 — Additional file 2. Supplementary Tables. Table S1: shNRF2 sequences. Table S2: Primer sequences. Table S3: List of antibodies. Table S4: Plasmids sequences. [file 12964_2022_979_MOESM3_ESM.docx]

**Supplementary Tables**

**Table S1.** shNRF2 sequences. **Table S2.** Primer sequences. **Table S3.** List of antibodies. **Table S4.** Plasmids sequences.

**Supplementary Table 1: shNRF2 sequences**

| shNRF2 | sense | antisense |
| --- | --- | --- |
| shNRF2 1# | GATCCCCGGCAT  TTCACTAAACAC  AACTCGAGTTGT  GTTTAGTGAAAT  GCCGGTTTTTG | AATTCAAAAACCGGCATTTCACTAAACACAACTCGAGTTGTGTTTAGTGAAATGCCGGG |
| shNRF2 2# | GATCCGCAGCAAA  CAAGAGATGGCAA  CTCGAGTTGCCATC  TCTTGTTTGCTGCT  TTTTG | AATTCAAAAAGCAG  CAAACAAGAGATGG  CAACTCGAGTTGCC  ATCTCTTGTTTGCTG  CG |

**Supplementary Table 2: Primer sequences**

|  | sequence（5'-3'） | |
| --- | --- | --- |
| NRF2 | F: CACATCCAGTCAGAAACCAGTG |  |
|  | R: GGAATGTCTGCGCCAAAAGCT |  |
| ERα | F: GCTTACTGACCAACCTGGCAGA |  |
|  | R: GGATCTCTAGCCAGGCACATTC |  |
| ERβ | F: AGAGTCCCTGGTGGTGTGAAGC |  |
|  | R: GACAGCGCAGAAGTGAGCATC |  |
| GPR30 | F: GCAGGTCCAGCAGAGGTACAC |  |
|  | R: GAGAGTGGGAAGAAACAGATG |  |
| CD44 | F: CCAGAAGGAACAGTGGTTTGGC |  |
|  | R: ACTGTCCTCTGGGCTTGGTGTT |  |
| CD49f | F: CGAAACCAAGGTTCTGAGCCCA |  |
|  | R: CTTGGATCTCCACTGAGGCAGT |  |
| GCLC | F: GGAAGTGGATGTGGACACCAGA |  |
|  | R: GCTTGTAGTCAGGATGGTTTGC |  |
| GCLM | F: TCTTGCCTCCTGCTGTGTGATG |  |
|  | R: TTGGAAACTTGCTTCAGAAAGC |  |
| TXNRD1 | F: GTTACTTGGGCATCCCTGGTGA |  |
|  | R: CGCACTCCAAAGCGACATAGG |  |
| ABCC2 | F: GCCAACTTGTGGCTGTGATAGG |  |
|  | R: ATCCAGGACTGCTGTGGGACAT |  |
| ABCB1 | F: GCTGTCAAGGAAGCCAATGCCT |  |
|  | R: TGCAATGGCGATCCTCTGCTTC |  |
| ABCG2 | F: GTTCTCAGCAGCTCTTCGGCTT |  |
|  | R: TCCTCCAGACACACCACGGATA |  |
| Bcl-2 | F: ATCGCCCTGTGGATGACTGAGT |  |
|  | R: GCCAGGAGAAATCAAACAGAG |  |
| AR | F: ATGGTGAGCCGAGTGCCCTATC |  |
|  | R: ATGGTCCCTGGCAGTCTCCAAA |  |
| HPRT | F: TGACACTGGCAAAACAATGCA |  |
|  | R: GGTCCTTTTCACCAGCAAGCT |  |
| NRF2 promotor ChIP | F: TGACTGCGAACACGAGCTG |  |
|  | R: TTTACCGCCCGAGAATGGCG |  |
| siERα 1# | UUCUCCGAACGUGUCACGUTT |  |
| siERα 2# | GAUGAAAGGUGGGAUACGATT |  |
| siNRF2 1# | CAGUCUUCAUUGCUACUAATT |  |
| siNRF2 2# | GAAUGGUCCUAAAACACCATT |  |
| siAR 1# | AAGACGCUUCUACCAGCUCAC |  |
| siAR 2# | GGAUUUGAGGUUACCUCAATT |  |
| siERβ 1# | GAGGGUACAAGUCCUCAAUTT |  |
| siERβ 2# | UCUUAAUCGCGUAUAAGGCTT |  |
| siGPR30 1# | GCUGUACAUUGAGCAGAAATT |  |
| siGPR30 2# | UAGGAAACCUCACGACUGGUU |  |
| Negative sequence | UUCUCCGAACGUGUCACGUTT |  |

**Supplementary Table 3: List of antibodies**

| antibody | application | antigen | dilution ratio | company |
| --- | --- | --- | --- | --- |
| ABCG2 | WB | Rabbit | 1:500 | Abcam,ab52897 |
|  | IF | Rabbit | 1:100 | Abcam,ab52897 |
| AR | WB | Rabbit | 1:500 | Dako，M3562 |
| Bcl-2 | WB | Rabbit | 1:500 | Abcam, ab59348 |
| CD44 | WB | Mouse | 1:500 | Abcam, ab6124 |
|  | IF | Mouse | 1:200 | Abcam, ab6124 |
| CD49f | WB | Mouse | 1:500 | Abcam, ab194969 |
|  | IF | Mouse | 1:200 | Abcam, ab194969 |
| ERα | WB | Mouse | 1:1000 | Santa, sc-542 |
|  | IF | Mouse | 1:100 | Santa, sc-542 |
|  | IHC | Rabbit | 1:500 | Abcam, ab32063 |
|  | ChIP | Rabbit | 2μl/100μl | Abcam, ab32063 |
| NRF2 | WB | Rabbit | 1:500 | Proteintech,16396-1-AP |
|  | IF | Rabbit | 1:100 | Proteintech,16396-1-AP |
|  | IHC | Rabbit | 1:200 | Proteintech,16396-1-AP |
| GADPH | WB | Mouse | 1:5000 | KANG CHEN, KC-5G4 |

Abbreviation: WB: Western Blot; IHC: Immunohistochemical; IF: Immunofluorescence.

**Supplementary Table 4: Plasmids sequences**

| Name | sequence |
| --- | --- |
| Normal-luc | GGTACCCGTTCAGGGTGACTGCGAACACGAGCTGCCGGAGCTGTCCACATCTCCCCTAGGCAGGGCCCACTGGCCCCAGCCCGGGAAGGGAGCAAGGGCGGGAGGGCTCGAG |
| Mutation-luc | GGTACCCGTTCAGGGTGACTGCGAACACGAGCTGCCGGAGCTGTCCACATCTCCCTAGGCAGGGCCCATCGGCCCCAGCCCGGGAAGGGAGCAAGGGCGGGAGGGCTCGAG |
