# Supplementary material for: The ERα-NRF2 signalling axis promotes bicalutamide resistance in prostate cancer
Source: Cell Commun Signal. 2022 Nov 14;20:178. doi: 10.1186/s12964-022-00979-0 (PMC9661764; doi:10.1186/s12964-022-00979-0)
Supplement: Supplementary file 4 — Additional file 3. Supplementary Figures. Fig. S1. (A) Representative IF graphs of NRF2 in LNCaP abl cells treated with DMSO or 10 nM E2 treatment; scale bar, 50 μm. (B) Western blot analysis showing LNCaP abl or PC3 with NRF2 knockdown. (C) qPCR analysis showing expression changes of the indicated genes in LNCaP abl and PC3 treated with either DMSO or 10 nM E2 (one-way ANOVA). All studies were repeated at least three times. The data are presented as the mean ± SEM. * P < 0.05, **P < 0.01. Fig. S2. (A) qPCR analysis of the indicated in LNCaP abl or PC3 cells with or without ICI182780 after treatment with either DMSO or 10 nM E2 (one-way ANOVA). (B) qPCR and Western blot analysis of LNCaP, LNCaP abl, and PC3 cells transfected with siAR and siCtrl. qPCR analysis showing expression changes of NRF2(t-test). (C) qPCR and Western blot analysis were conducted in LNCaP abl and PC3 cells transfected with siERα, siERβ, siGPR30 and siCtrl. qPCR analysis showing expression changes of NRF2(t-test). All studies were repeated at least three times; the values are the mean ± SEM; *P < 0.05, **P < 0.01. Fig. S3. Scratch test was conducted in PC3 cells with different treatments, scale bar, 200 μm. All studies were repeated at least three times; the values are the mean ± SEM; *P < 0.05. Fig. S4. The mRNA-seq analysis indicated that the expression of antiapoptotic genes was up-regulated in bicalutamide resistant cells and positively correlated with the expression of NRF2. The heatmap was generated by an online analytic tool (www.xiantao.love). [file 12964_2022_979_MOESM4_ESM.docx]

**
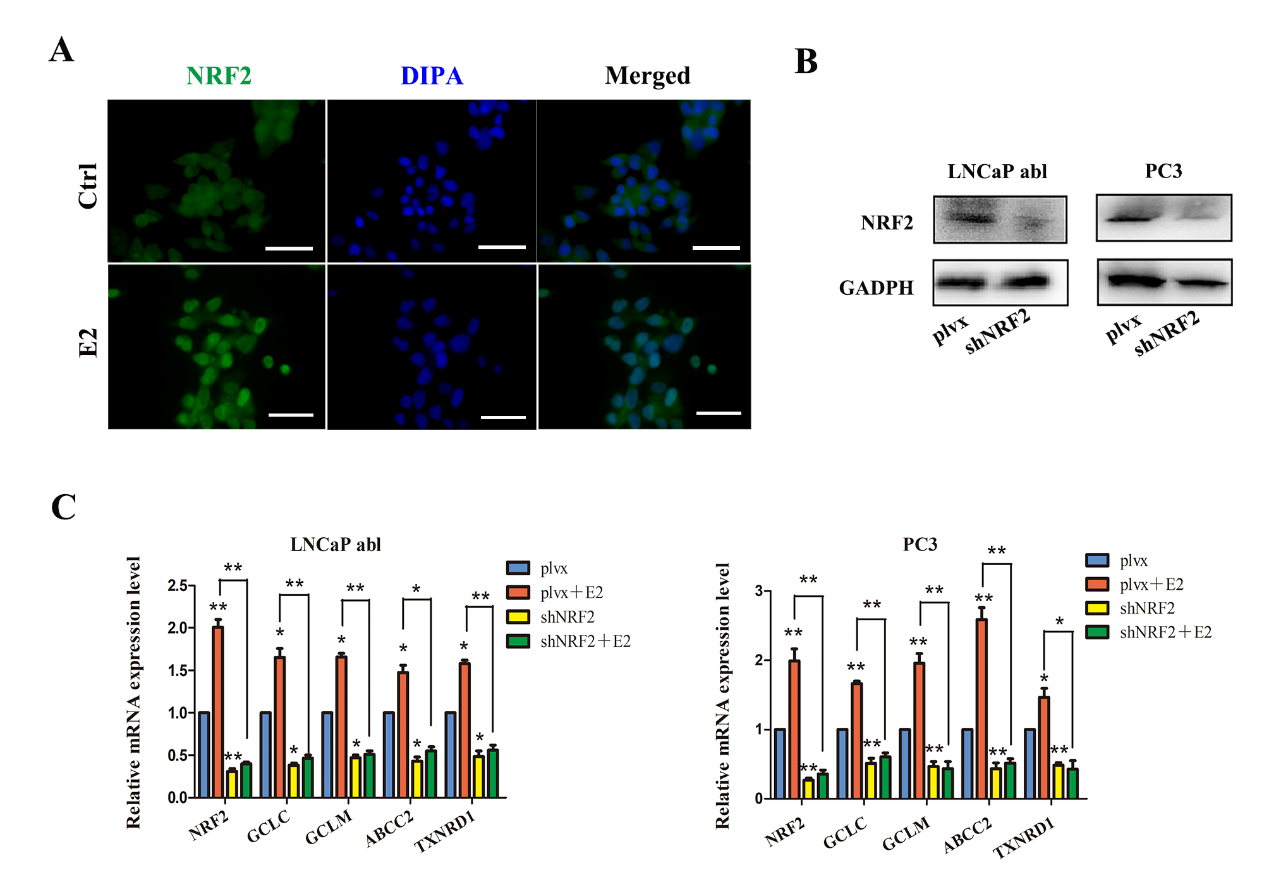
Fig.S1.** (A) Representative IF graphs of NRF2 in LNCaP abl cells treated with DMSO or 10 nM E2 treatment; scale bar, 50 μm. (B) Western blot analysis showing LNCaP abl or PC3 with NRF2 knockdown. (C) qPCR analysis showing expression changes of the indicated genes in LNCaP abl and PC3 treated with either DMSO or 10 nM E2 (one-way ANOVA). All studies were repeated at least three times. The data are presented as the mean ± SEM. * P < 0.05, **P < 0.01.


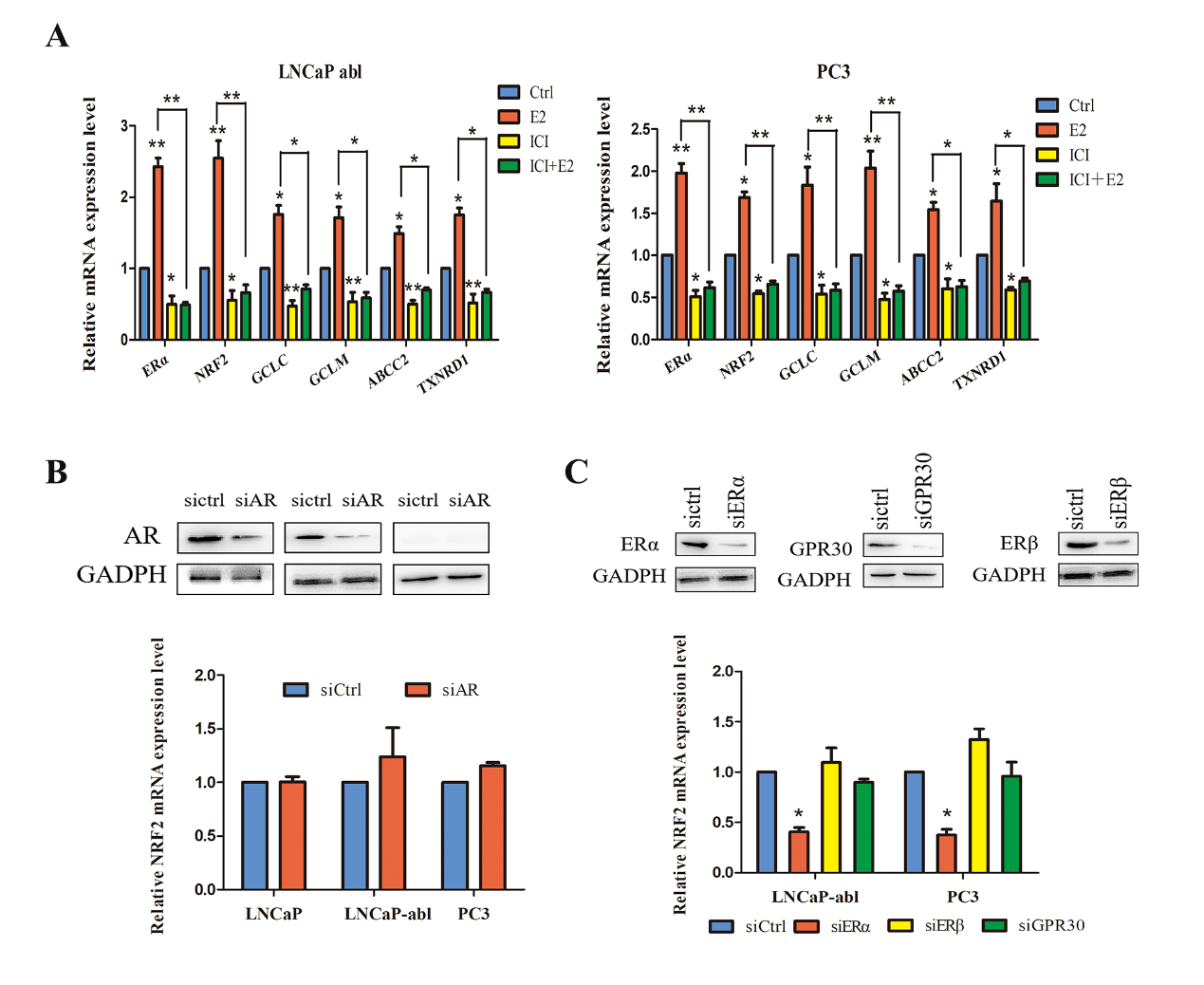


Fig. S2. (A) qPCR analysis of the indicated in LNCaP abl or PC3 cells with or without ICI182780 after treatment with either DMSO or 10 nM E2 (one-way ANOVA). (B) qPCR and Western blot analysis of LNCaP, LNCaP abl, and PC3 cells transfected with siAR and siCtrl. qPCR analysis showing expression changes of NRF2(t-test). (C) qPCR and Western blot analysis were conducted in LNCaP abl and PC3 cells transfected with siERα, siERβ, siGPR30 and siCtrl. qPCR analysis showing expression changes of NRF2(t-test). All studies were repeated at least three times; the values are the mean ± SEM; *P < 0.05, **P < 0.01.


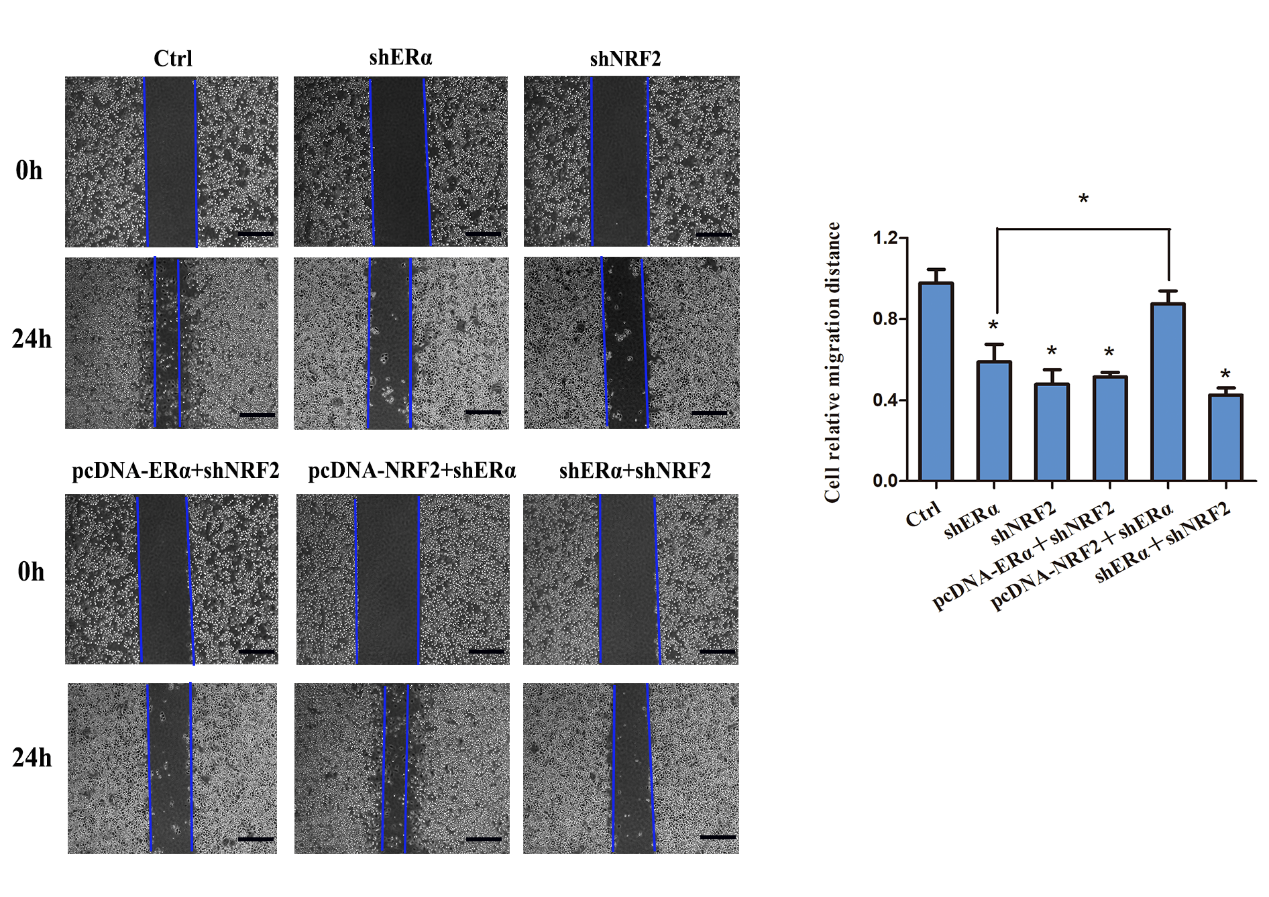


Fig. S3. Scratch test was conducted in PC3 cells with different treatments, scale bar, 200 μm. All studies were repeated at least three times; the values are the mean ± SEM; *P < 0.05.


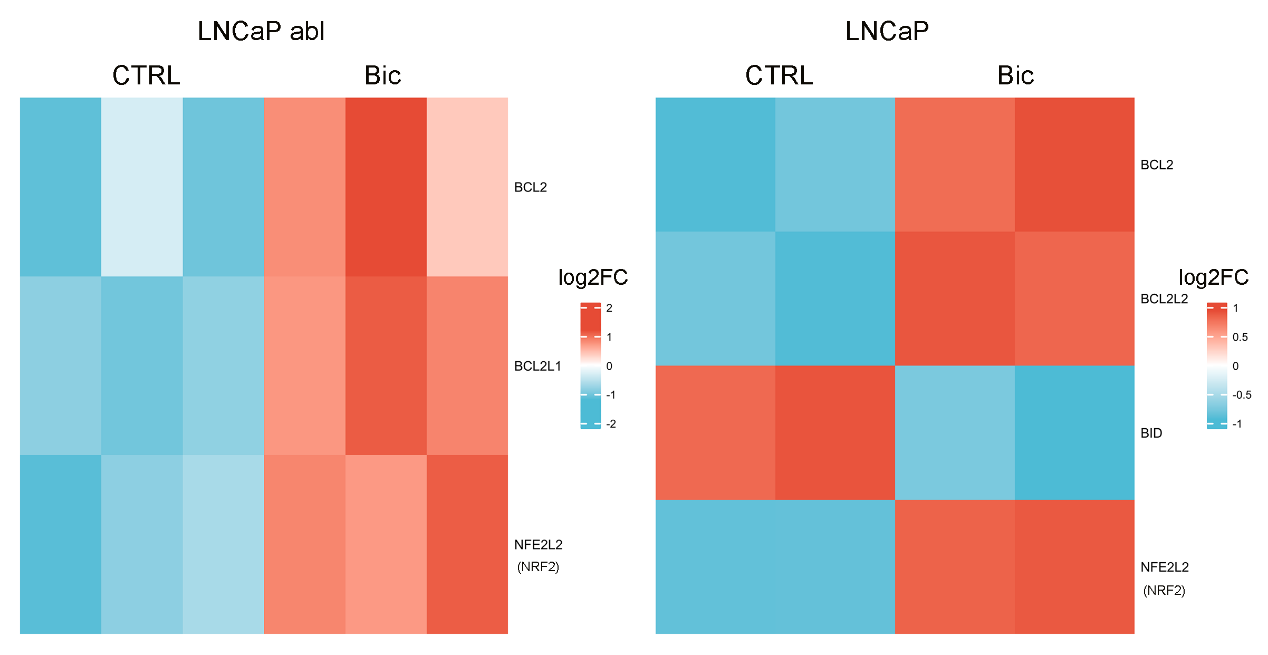


Fig. S4. The mRNA-seq analysis indicated that the expression of antiapoptotic genes was up-regulated in bicalutamide resistant cells and positively correlated with the expression of NRF2. The heatmap was generated by an online analytic tool ([www.xiantao.love](http://www.xiantao.love)).
